# Change in Age Pattern of Persons with Dengue, Northeastern Brazil

**DOI:** 10.3201/eid1701.100321

**Published:** 2011-01

**Authors:** Luciano P. Cavalcanti, Dina Vilar, Reinaldo Souza-Santos, Maria G. Teixeira

**Affiliations:** Author affiliations: Federal University of Ceará, Fortaleza, Brazil (L.P. Cavalcanti);; Health Secretariat of Ceará State, Fortaleza (D. Vilar);; National School of Public Health, Rio de Janeiro, Brazil (R. Souza-Santos);; Federal University of Bahia, Salvador, Brazil (M.G. Teixeira)

**Keywords:** Dengue, dengue fever, dengue hemorrhagic fever, viruses, epidemiology, age shift, Brazil, letter

**To the Editor:** Approximately 40% of the world’s population is at risk for dengue (*1*). Epidemiologic characteristics of dengue differ by region, and disease incidence varies by patient age. In Southeast Asia, incidence of dengue fever (DF) and dengue hemorrhagic fever (DHF) is highest among children (*2**,**3*); in the Western Hemisphere, incidence of these diseases is higher among adults.

In Brazil, which has ≈80% of dengue cases in the Western Hemisphere, adults are at risk for dengue virus (DENV) infection (*3**,**4*). However, in 2007, a total of 53% of persons in Brazil hospitalized with DHF were children <15 years of age; this proportion was highest (65.4%) in children in northeastern Brazil (*5*).

In Ceará, a state in northeastern Brazil, DENV-1 epidemics have occurred since 1987. DHF cases have been reported since 1994 when DENV-2 was identified. In 2003, a severe DENV-3 epidemic occurred, and DHF incidence was high among adults (*6*). However, since 2007, incidence of DENV infection has been highest among children (*7*). To better understand factors that could affect this change in risk by age group, we studied the temporal progression of age distribution of persons with dengue during 1998–2008 in Ceará.

We used data for Ceará from the National System of Notifiable Diseases (DF and DHF cases), the Hospital Admission Data System (dengue hospitalizations) (*8*), and the Central Public Health Laboratory (virus isolation). For each age group (<10, 10–19, 20–59, and >60 years), we calculated incidence of DF and hospitalization rate for DHF. We also calculated proportions of dengue serotypes per year (2001–2008). Medians for continuous variables were compared by using the Kruskal-Wallis test. Analyses were performed by using Epi Info version 6.0 software (Centers for Disease Control and Prevention, Atlanta, GA, USA).

From 1998 (10.8 cases/100,000 persons) through 2007 (236.7 cases/100,000 persons), DF incidence was lowest among persons <10 years of age. However, the incidence was highest (599.4 cases/100,000 persons) for this age group in 2008. In 2007, incidence among persons <10 years of age (236.7 cases/100,000 persons) was similar to that among persons 10–19, 20–59, and >60 years of age (305.6, 331.5, and 249.9 cases/100,000 persons, respectively).

Since 2007, the incidence of DHF among children was already higher (4,884 cases/100,000 persons) than among the other age groups (3,261, 3,387 and 2,789 cases/100,000 persons, respectively). In 2008, incidence of DHF among children was 8,992 cases/100,000 persons, which was 2× that among persons 10–19 and 20–59 years of age and >3× that among persons >60 years of age. Median age of persons with DHF decreased from 38 years in 2001 to 18 years in 2008 (p<0.0001). Children <10 years age, who in 2001 accounted for 5% of all cases, accounted for 33% of cases in 2008.

The hospitalization rate for dengue among children in Ceará followed a pattern similar to that for DHF and increased for children <10 years of age. In 2008, this pattern was greater for this age group (1.449/1,000 hospitalizations) than in any other age group. DENV-2 (52.3%) and DENV-1 (47.7%) were co-circulating in 2002. DENV-3 was isolated in 2003 and represented >40% of isolations. At this time, DENV-2 and DENV-1 represented 7.4% and 48.5% of isolations, respectively. DENV-3 then predominated in Ceará until 2006 when DENV-2 reemerged (1.4%). DENV-2 became the predominant serotype in 2007 (84%) and 2008 (76.1%) (Table).

The increase in DHF incidence among children in Ceará during 2007–2008 was greater than the overall increase in Brazil (*4**,**5*). Because the predominant serotype in Ceará in 2007–2008 was DENV-2, two hypotheses may explain this phenomenon.

First, a more virulent DENV-2 may have been introduced. Genetic sequencing of DENV-2 circulating in another state in Brazil during the 2008 epidemic, compared with the 1990 and 1998 epidemics, showed that all isolates had the same genotype (American/Asiatic); only a 2% had a phylogenetic change (*9*). Such a small difference cannot explain this change in the age group affected by dengue.

Second, the time when 3 serotypes circulated in Ceará may not have favored development of antibodies against DENV-3 in children <10 years of age, although they were susceptible to DENV-2. DENV-1 and DENV-2 were circulating in Ceará before 2002 and caused DF epidemics and a few DHF cases. However, these diseases occurred predominantly in adults. Conversely, children had little likelihood of being infected with DENV-2 because the incidence of dengue before 2002 was low and the 2000 birth cohort had little contact with DENV-2, which was no longer circulating. Thus, most persons susceptible to DENV-2 were children.

DENV-3 was circulating during 2003–2006 and affected persons of all ages. Thus, when DENV-2 reemerged in 2006, many adults in Ceará already had antibodies against it. However, children had no antibodies against DENV-2, although some had antibodies against DENV-3. This immunologic difference may have caused the higher incidence of dengue among children in Ceará during 2007–2008, particularly in view of severity of the 2 epidemics, increased risk for DHF (*10*), and number of hospitalizations for dengue during this period.

**Table:** Confirmed cases of dengue fever and dengue hemorrhagic fever, Ceará, Brazil, 1998–2008*

Characteristic	1998	1999	2000	2001	2002	2003	2004	2005	2006	2007	2008
No. DF cases	3,581	9,757	13,645	34,390	16,465	23,796	3,094	22,817	25,569	25,026	44,508
No. DHF cases	4	3	4	82	72	292	14	199	173	280	408
Serotype of DENV isolated, %											
1	–	–	–	47.7	48.5	1.9	0	2.5	0	0	0
2	–	–	–	52.3	7.4	1.9	0	0.0	1.4	84.0	76.1
3	–	–	–	0	44.1	96.2	100.0	97.5	98.6	16.0	23.9
Incidence of DF and DHF by age, y†											
<10	10.8	37.8	65.4	174.7	78.5	128.0	14.3	126.5	116.0	236.7	599.4
10–19	26.7	95.4	129.3	321.4	160.6	250.4	34.3	198.2	247.9	305.6	574.4
20–59	68.3	198.7	263.6	659.3	304.3	416.9	53.6	365.2	412.6	331.5	521.9
>60	143.1	210.0	194.8	423.4	223.3	313.0	39.1	441.5	422.2	249.9	301.0
Incidence of DHF by age, y†											
<10	0.000	0.000	0.000	0.236	0.114	1.423	0.068	1.011	0.890	4.884	8.992
10–19	0.062	0.000	0.059	0.796	0.449	3.601	0.065	2.512	1.771	3.261	4.968
20–59	0.064	0.094	0.058	1.361	1.474	4.655	0.318	3.074	3.053	3.387	4.358
>60	0.174	0.000	0.152	1.890	1.289	5.134	0.000	2.650	0.832	2.789	2.868
Hospitalizations for dengue (classic and hemorrhagic) by age, y‡											
<10	0.008	0.014	0.015	0.050	0.145	0.352	0.073	0.395	0.266	0.667	1.449
10–19	0.006	0.010	0.020	0.131	0.255	0.632	0.156	0.646	0.497	0.750	1.128
20–59	0.005	0.011	0.024	0.256	0.440	0.847	0.255	0.804	0.572	0.710	0.786
>60	0.009	0.009	0.021	0.401	0.658	1.159	0.389	1.012	0.664	0.954	0.922

*Source: Hospitalization Information System, Notifiable Disease Information System, and Central Public Health Laboratory of Ceará. DF, dengue fever; DHF, dengue hemorrhagic fever, DENV, dengue virus; –, no information. †Per 100,000 persons. ‡Per 1,000 persons.
